# Long-Term Oncological Outcomes After Laparoscopic Resection and Thermal Ablation of Colorectal Liver Metastases Within a Parenchyma-Sparing Strategy

**DOI:** 10.1245/s10434-026-19621-1

**Published:** 2026-04-14

**Authors:** I. Schrøder Hansen, D. L. Aghayan, E. Pelanis, B. I. Røsok, K. W. Brudvik, S. Yaqub, K. Lassen, I. Bjørk, K. Brabrand, B. Edwin, T. Syversveen, Å. A. Fretland

**Affiliations:** 1 The Intervention Centre, Oslo University Hospital, Oslo, Norway; 2 Department of HPB-Surgery, Oslo University Hospital, Oslo, Norway; 3 Institute of Clinical Medicine, University of Oslo, Oslo, Norway; 4 Department of Surgery, Vestre Viken Hospital Trust, Ringerike Hospital, Hønefoss, Norway; 5 Department of Surgery N1, Yerevan State Medical University after M. Heratsi, Yerevan, Armenia; 6 Institute of Clinical Medicine, UiT the Arctic University of Norway, Tromsø, Norway; 7 Department of Radiology, Oslo University Hospital, Oslo, Norway

**Keywords:** Colorectal liver metastases, Laparoscopic liver resection, Thermal ablation, Parenchymal sparing

## Abstract

**Background:**

Thermal ablation of colorectal liver metastases (CRLM) is traditionally reserved for patients who are not candidates for surgical resection, but it is increasingly applied as a parenchyma-sparing alternative. This study investigates long-term outcomes following laparoscopic resection and thermal ablation in a parenchyma-sparing treatment strategy.

**Methods:**

Cohort study of patients with CRLM treated with laparoscopic resection or thermal ablation at Oslo University Hospital between 2007 and 2020, identified from two prospectively maintained databases. The primary endpoint was the average treatment effect on survival, estimated using inverse probability weighting to adjust for prognostic variables. Secondary endpoints included overall survival and progression-free survival. In case of recurrence of liver metastases after the study treatment, both ablation and resection were used.

**Results:**

A total of 207 ablation procedures and 499 resection procedures were eligible for analysis. Five-year overall survival was 55.2% in the ablation group and 55.1% in the resection group. The estimated average treatment effect of ablation versus resection was − 0.34 months (95% confidence interval − 7.29 to 6.57; *P* = 0.919), indicating that treating all patients with ablation would yield an average survival of 0.34 months shorter than treating all patients with resection.

**Conclusions:**

In a parenchymal-sparing paradigm, thermal ablation yields long-term survival comparable to resection for CRLM.

**Supplementary Information:**

The online version contains supplementary material available at 10.1245/s10434-026-19621-1.

Colorectal cancer is the third most commonly diagnosed cancer worldwide and the second leading cause of cancer-related mortality.^1^ Between 25 and 50% of patients develop colorectal liver metastases (CRLM) during the course of their disease.^2^ Historically, liver resection has been the gold standard for treatment of CRLM and considered the only potentially curative treatment option, whereas thermal ablation has traditionally been reserved for patients where resection is not an option.

Previous comparative studies have reported inferior overall survival (OS) in patients treated with ablation,^3^ but these findings were hampered by selection bias. In recent years, there have been advances in ablation technology and techniques, and studies adjusting for selection bias report no difference in OS.^4,5^ Nevertheless, high rates of local tumour progression remain a concern.^6^

The recently published randomized controlled COLLISION trial reported non-inferior OS for thermal ablation versus resection,^7^ supporting the broader use of ablation beyond nonresectable settings. At Oslo University Hospital, ablation has been integrated into a parenchyma-sparing strategy. Treatment modality has been determined through multidisciplinary tumour-board (MDT) meetings where ablation has been considered noninferior to resection for lesions ≤ 3 cm, despite absence of high-quality data on the efficacy of ablation in resectable tumours. Initially, ablation was considered noninferior primarily for small, deep-seated lesions in which resection would have caused substantial parenchymal loss, but this was gradually extended to all lesions ≤3 cm that were amenable to ablation. This institutional approach provides a unique opportunity to evaluate long-term outcomes of ablation and resection in a real-world parenchyma-sparing framework.

Most prior studies compare only the initial treatment for CRLM, despite the high incidence of hepatic recurrence and the frequent need for repeated local treatment.^8,9^ Modern multimodal treatment frequently alternates between using resection, ablation, and systemic oncologic therapy. Thus, a patient with recurrent disease will often have been treated with multiple different modalities during their disease trajectory. This study investigates outcomes after resection and ablation of CRLM at any point in the patient’s disease trajectory within a parenchyma-sparing strategy. The hypothesis of this study is that thermal ablation does not lead to inferior survival compared to resection, when adjusted for selection bias.

## Methods

This was a cohort study of patients with CRLM who underwent thermal ablation or laparoscopic liver resection at Oslo University Hospital between January 2007 and June 2020. Procedures for radically treatable cancer recurrence within this timeframe were included. Approval for this study was granted by the Data Protection Officer at Oslo University Hospital (approval reference 25/25046) with a waiver for informed consent.

Procedures that combined ablation and resection, as well as those involving other simultaneous interventions, were excluded. Furthermore, any procedures related to patients treated as part of a two-stage liver surgery protocol, and procedures involving tumours larger than 3 cm, were excluded.

Patients with extrahepatic metastases were not excluded. Extrahepatic disease was generally limited to lung and lymph-node metastases in the hepatic hilum, as all procedures were performed with curative intent. In rare cases, highly selected patients with peritoneal, adrenal, or extra-hilar lymph-node metastases were also accepted for local treatment for CRLM, provided that these extrahepatic lesions had exhibited a durable response to systemic therapy and were planned for curative-intent treatment.

The primary endpoint was the estimated average treatment effect (ATE) on overall survival following inverse probability weighting (IPW). By weighting each observation with the inverse probability of receiving the treatment actually administered, IPW balances baseline covariates across the two groups, thereby approximating the conditions of a randomized trial and allowing a fair comparison of outcomes.^10^ Analyses were performed per procedure, and patients could appear in both treatment groups if treated with more than one modality within this timeframe.

Secondary endpoints were overall survival and progression-free survival. To avoid counting any patient more than once in the secondary endpoint survival curves, only the first qualifying procedure per patient was retained for those analyses.

The protocol outcomes were defined post hoc. Relevant data were extracted from two prospectively maintained databases—one for thermal ablation procedures and the other for laparoscopic liver resections—and were supplemented by patients’ medical records. Local tumour progression after ablation was evaluated by a radiologist experienced in liver ablations. Local tumour progression was defined as new tumour tissue directly adjacent (0 mm) to the ablation zone at the earliest time point at which the new tumour is detected. This definition represents a modified version of the updated standardized terminology.^11^

The decision to select ablation or resection as treatment modality was made during MDT meetings involving oncologists, liver surgeons, and interventional radiologists experienced in ablation techniques. Ablations were performed under ultrasound guidance, and margins were assessed intra-procedurally using contrast-enhanced ultrasound. A CT scan was routinely obtained the day after the procedure to serve as a baseline for follow-up. Ablation confirmation software was not used. Resections were performed with parenchyma-sparing intent, using surgical technique described previously.^12,13^ Patients were followed according to national guidelines, with identical surveillance in both groups: CT imaging of the chest and abdomen every 6 months for 5 years after the last liver intervention.

### Statistical Analysis

Continuous data were presented as means with standard deviations (SD) or medians with interquartile ranges (IQR), while categorical data were reported as frequencies and percentages. Differences between groups were analysed by using Pearson’s chi-squared test, linear regression or the Kruskal-Wallis test, as appropriate. Analyses were performed by using Stata version 18 (StataCorp, College Station, TX).

Cox regression analysis was performed to evaluate factors influencing survival. Missing covariate data in the Cox regression analysis were addressed by multiple imputation using chained equations in Stata, generating 25 imputed datasets. Imputation models consisted of linear regression for continuous variables, logistic regression for binary variables, multinomial logistic regression for nominal categorical variables, and ordered logistic regression for ordinal variables.

Inverse probability weighting was performed to balance baseline characteristics between treatment groups. After weighting, the ATE on survival was estimated by comparing the estimated mean outcomes of the two groups. The ATE and potential-outcome means analysis was performed using baseline and prognostic variables (age, gender, body mass index, American Society of Anesthesiologists score, positive CEA, primary tumor nodal status and sidedness, diagnosis of first CRLM within 12 months after primary tumour, previous liver surgery, neoadjuvant chemotherapy, date of operation, time from primary tumour to study treatment, maximal liver tumor size, number of liver tumours, and extrahepatic metastases at time of study treatment). For analysis of ATE, the Stata command effects with the IPW method was used. Patients with missing data were excluded from the analysis of ATE.

Repeated treatments for hepatic recurrence that occurred during the study period were included in the analysis of ATE and in the multivariable Cox regression. To account for repeated treatments in the same patient, we employed robust standard errors clustered by patient identifier.

Kaplan–Meier survival curves for OS and PFS were created using the first qualifying treatment for each patient. Differences between groups were compared with the log-rank test.

## Results

A total of 499 laparoscopic resection procedures and 206 thermal ablation procedures meeting the inclusion criteria were identified during the study period. Median follow-up was 50.4 months (IQR 28.4–72.5).

The procedures were divided into two time intervals: 2007–2013 and 2014–2020. In the latter interval, procedure volumes increased for both thermal ablation and laparoscopic resection, with a proportionally greater rise observed in ablation procedures (Table 1).

**Table 1:** Baseline characteristics (all procedures)

	Resection (*N* = 499)	Ablation (*N*= 206)	*P*
*Time interval*			
2007–2013	189 (37.9%)	49 (23.8%)	<0.001
2014–2020	310 (62.1%)	157 (76.2%)	
*Patient characteristics*			
Age, years, mean (SD)	65.57 (11.13)	64.06 (11.78)	0.106
Male gender	290 (58.9%)	130 (63.1%)	0.219
BMI, kg/m^2^, mean (SD)	25.21 (4.34)	25.22 (4.17)	0.975
*ASA score*			
1	16 (3.2%)	1 (0.5%)	0.006
2	296 (60%)	102 (52.6%)	
3	175 (35.5%)	91 (46.9%)	
4	6 (1.2%)	0 (0%)	
*Primary tumour characteristics*			
Right-sided primary tumour	81 (21.6%)	41 (19.9%)	0.631
Positive nodal status	220 (61.1%)	126 (66%)	0.262
*Disease course and oncological treatment*			
First liver metastasis within 12 months after primary tumour	286 (64.9%)	164 (79.6%)	<0.001
Time from surgery primary tumour to study treatment, months, median (IQR)	9.81 (3.43–21.14)	13.18 (5.36–25.2)	0.003
Study procedure before surgery primary tumour (“liver first”)	44 (8.8%)	10 (4.9%)	0.072
Neoadjuvant chemotherapy	197 (42.2%)	90 (43.9%)	0.678
Extrahepatic metastases at time of study treatment	96 (22.8%)	34 (16.5%)	0.068
CEA, µg/L, median (IQR)	4 (2.2–9.2)	3.6 (2.4–8.5)	0.467
*Liver tumour characteristics*			
Number of lesions, mean (SD)	1.43 (0.84)	1.38 (0.89)	0.501
Largest tumour size, mm, mean (SD)	17.7 (7.08)	15.3 (6.76)	<0.001
*Previous liver-directed treatments*			
0	371 (75.1%)	100 (48.5%)	<0.001
1	101 (20.4%)	56 (27.2%)	
2	17 (3.4%)	32 (15.5%)	
3	5 (1%)	11 (5.3%)	
4	0 (0%)	4 (1.9%)	
5	0 (0%)	3 (1.5%)	

*SD* standard deviation; *ASA* American Society of Anesthesiologists Physical Status; *IQR* interquartile range; *CEA* carcinoembryonic antigen

Compared with patients treated with liver resection, those treated with ablation had higher rate of diagnosis of first CRLM within 12 months after primary tumour, a longer interval from surgery of primary tumour to the study treatment, more prior liver-directed procedures and higher ASA scores. Patients treated with resection had larger maximal tumour size. Data on ethnicity was not available in the relevant databases.

The ablations were mainly performed percutaneously, with the reminder performed open; no conversions occurred. All resections were initiated laparoscopically, with a low rate of conversion to either hand-assisted laparoscopy or laparotomy. Patients treated with resection had longer postoperative hospital stay and fewer new liver treatments for recurrence during follow-up. There was no difference in overall complication rate, but the resection group had a higher rate of complications Accordion ≥ 3, including three deaths (Table 2).

**Table 2:** Results (all procedures)

	Resection (*N* = 499)		Ablation (*N* = 206)		*P*
*Procedural access*					
Percutaneous	0 (0%)		191 (92.7%)		
Laparoscopy	498 (99.8%)		0 (0%)		
Hand-assisted laparoscopy	1 (0.2%)		0 (0%)		
Open	0 (0%)		15 (7.3%)		
*Conversion of access*					
Hand-assisted laparoscopy	11 (2.2%)		0 (0%)		0.022
Open	7 (1.4%)		0 (0%)		
*Ablation method*					
RFA			175 (85%)		
MWA			31 (15%)		
Postoperative hospital stay, days, mean (SD)	3 (4.23)		1.71 (1.66)		<0.001
*Complications*					
Accordion grade					
0	432 (86.7%)		189 (91.7%)		0.089
1	9 (1.8%)		4 (1.9%)		
2	28 (5.6%)		10 (4.9%)		
3	18 (3.6%)		1 (0.5%)		
4	8 (1.6%)		1 (0.5%)		
5	0 (0%)		1 (0.5%)		
6	3 (0.6%)		0 (0%)		
Accordion ≥3	29 (5.8%)		3 (1.5%)		0.011
*New liver procedures during follow-up*					
0	381 (77.8%)		143 (69.4%)		0.001
1	88 (18%)		37 (18%)		
2	18 (3.7%)		20 (9.7%)		
3	3 (0.6%)		4 (1.9%)		
4	0 (0%)		2 (1%)		

*RFA* radiofrequency ablation; *MWA* microwave ablation; *SD* standard deviation; *Accordion* Accordion Severity Grading System for surgical complications

### Local Tumour Progression After Ablation

Local tumour progression was observed in 46/206 (22.3%) ablations on procedural basis. Among patients whose largest tumour measured 0–20 mm, local tumour progression was observed after 22/152 (14.5%) ablation procedures compared with 24/54 (44.4%) among patients whose largest tumour measured 21–30 mm (*P* < 0.001). Data for local recurrence after resection were not available for this study.

### Inverse Probability Weighting and Average Treatment Effect

**Fig. 1:**
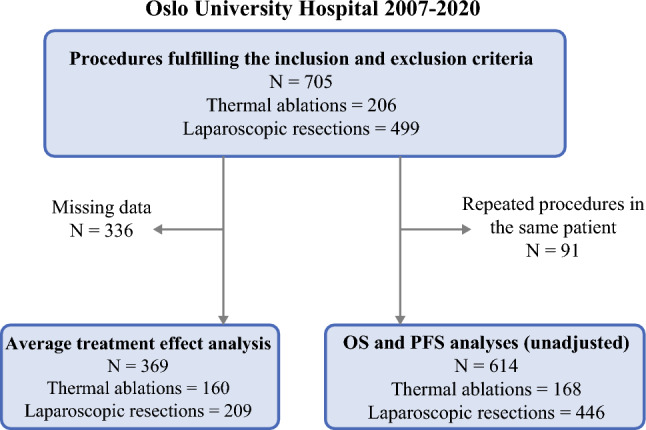
Flowchart of patients in analyses of ATE, OS and PFS. *ATE* average treatment effect; *OS* overall survival; *PFS* progression-free survival. The ATE analysis required complete data for each patient, and patients with missing data were therefore excluded. The unadjusted OS/PFS analyses could not account for repeated procedures in the same patient, so such procedures were excluded. Created in BioRender. Hansen, I. S. (2026) https://BioRender.com/5qdxclg

A total of 336 patients had missing data and were excluded from the ATE analysis (Fig. 1). Using inverse probability weighting, the estimated average treatment effect of ablation was − 0.34 months (95% CI − 7.29 to 6.57; *P* = 0.919), indicating that treating all patients with ablation would yield an average survival of 0.34 months shorter than treating all patients with resection.

### Cox Multivariate Analysis of Overall Survival

In a multivariate Cox regression, treatment modality was not associated with OS. Independent predictors of poorer overall survival included older age, positive nodal status of the primary tumour, receipt of neoadjuvant chemotherapy, higher number of liver metastases, larger maximal tumour diameter, presence of extrahepatic metastases, and a higher number of previous liver-directed treatments (Table 3).

**Table 3:** Cox multivariate regression analysis for overall survival (all procedures)

	HR	95% CI	*P*
Ablation	0.94	0.695–1.271	0.687
Time period 2014–2020	0.772	0.588–1.015	0.064
*Patient characteristics*			
Age, years	1.013	1.001–1.026	0.034
Male gender	1.056	0.8–1.394	0.702
BMI	1.004	0.968 –1.040	0.844
*ASA score*			
2	0.6	0.239–1.505	0.276
3	0.892	0.349–2.275	0.81
4	0.943	0.308–2.897	0.919
*Primary tumour characteristics*			
Right-sided primary tumour	1.228	0.872–1.73	0.24
Positive nodal status	1.403	1.012–1.947	0.042
*Disease course and oncological treatment*			
First liver metastasis within 12 months after primary tumour	0.801	0.574–1.117	0.19
Time from surgery primary tumour to study treatment	0.991	0.981–1.000	0.059
Neoadjuvant chemotherapy	1.503	1.141–1.979	0.004
Extrahepatic metastases at time of study treatment	1.474	1.091–1.977	0.011
CEA >5 µg/L	1.083	0.845–1.39	0.528
Number of previous liver-directed treatments	1.395	1.135–1.714	0.002
*Liver tumour characteristics*			
Number of lesions	1.195	1.071–1.332	0.001
Largest tumour size, mm	1.035	1.016–1.053	0

*HR* hazard ratio; *CI* confidence interval; *BMI* body mass index; *ASA* American Society of Anesthesiologists classification; *CEA* carcinoembryonic antigen

### Overall and Progression-free Survival (Unadjusted)

**Fig. 2:**
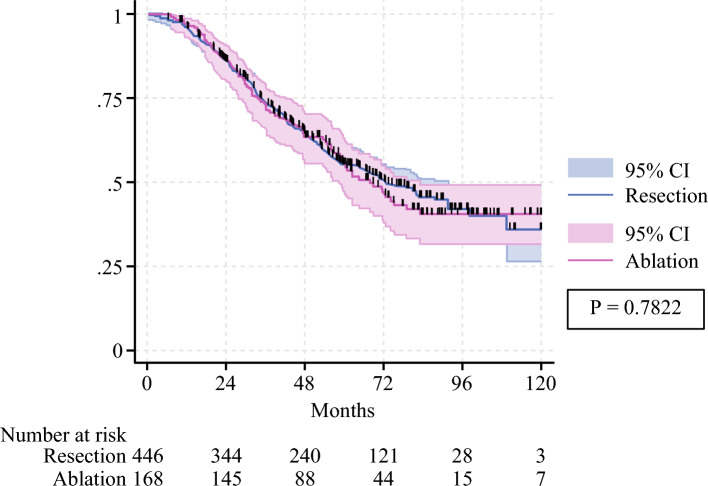
Overall survival after patient’s first liver treatment during the study interval

**Fig. 3:**
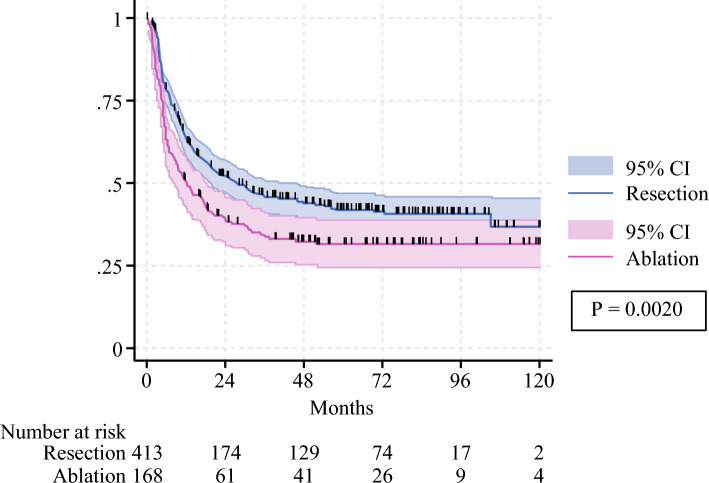
Progression-free survival after patient’s first liver treatment during the study interval

For the analyses of overall survival and progression-free survival, only the first qualifying procedure for each patient was included. This constituted 446 resection procedures and 168 ablation procedures. Baseline differences between groups remained consistent, except that extrahepatic metastases were less frequent in the ablation group (Table 4). Overall survival did not differ between groups; 5 year OS was 55.1% (95% CI 50–59.9%) after resection and 55.2% (95% CI 46.9–62.8%) after ablation (Fig. 2). Median OS was 72.6 (95% CI 59.9–91.7) months in the resection group and 67.7 (95% CI 58.4–83.1) months in the ablation group. 5-year progression-free survival was 41.9% (95% CI 36.7–46.9%) following resection and 31.4% (95% CI 24.4–38.7%) following ablation (Fig. 3). A sensitivity analysis restricted to patients with no prior liver-directed therapy similarly showed no difference in OS between groups (Supplementary Fig. 1). Subgroup analyses according to tumour size (0–20 mm and 21–30 mm) revealed no significant difference in OS between resection and ablation but demonstrated a significant difference in PFS in both subgroups (Supplementary Figs. 2 and 3).

**Table 4:** Baseline characteristics (first qualifying procedure per patient)

	Resection (*N* = 446)	Ablation (*N* = 168)	*P*
*Time interval*			
2007–2013	168 (37.7%)	39 (23.2%)	<0.001
2014–2020	278 (62.3%)	129 (76.8%)	
*Patient characteristics*			
Age, years, mean (SD)	65.99 (10.99)	64.72 (11.27)	0.205
Male gender	257 (57.6%)	104 (61.9%)	0.337
BMI, kg/m^2^, mean (SD)	25.29 (4.38)	25.3 (4.3)	0.987
*ASA*			
1	16 (3.6%)	1 (0.6%)	0.019
2	265 (60.2%)	84 (53.5%)	
3	154 (35%)	72 (45.9%)	
4	5 (1.1%)	0 (0%)	
*Primary tumour characteristics*			
Right-sided primary tumour	76 (22.7%)	32 (19%)	0.349
Positive nodal status	198 (60.9%)	107 (69.5%)	0.069
*Disease course and oncological treatment*			
First liver metastasis within 12 months after primary tumour	265 (64.6%)	132 (78.6%)	0.001
Time from surgery primary tumour to study treatment, months, median (IQR)	8.44 (2.76–20.39)	11.14 (4.8–20.96)	0.015
Study procedure before surgery primary tumour (“liver first”)	43 (9.6%)	10 (6%)	0.147
Neoadjuvant chemotherapy	181 (42.8%)	80 (47.6%)	0.286
Extrahepatic metastases at time of study treatment	87 (22.4%)	24 (14.3%)	0.028
CEA, µg/L, median (IQR)	4.0 (2.1–9)	3.6 (2.2–8.5)	0.346
*Liver tumour characteristics*			
Number of lesions, mean (SD)	1.45 (0.88)	1.39 (0.93)	0.442
Largest tumour size, mm, mean (SD)	17.72 (7.11)	15.24 (6.75)	<0.001
*Previous liver-directed treatments*			
0	366 (83%)	100 (59.5%)	<0.001
1	68 (15.4%)	41 (24.4%)	
2	6 (1.4%)	18 (10.7%)	
3	1 (0.2%)	6 (3.6%)	
4	0 (0%)	2 (1.2%)	
5	0 (0%)	1 (0.6%)	

*SD* standard deviation; *ASA* American Society of Anesthesiologists Physical Status; *IQR* interquartile range; *CEA* carcinoembryonic antigen; *BMI* body mass index

## Discussion

This study demonstrates that thermal ablation, when incorporated in a parenchyma-sparing multimodal strategy, yields long-term survival comparable to laparoscopic resection for CRLM. Survival outcomes remained similar after adjustment for baseline differences using inverse probability weighting, reinforcing previous evidence that ablation is noninferior to resection when selection bias is appropriately addressed.^4,5,7^

The findings contrast from previous studies, in that unadjusted analysis of OS was similar between the groups. This likely reflects the strategy of choosing the parenchyma-sparing treatment option, which leads to the groups being seemingly prognostically similar. However, the result might reflect modern multimodal treatment of CRLM, in which patients can be treated with multiple modalities over the course of their disease and the cumulative effect of all modalities determines survival outcomes.

A higher recurrence rate was observed after ablation, most likely reflecting differences in local tumour progression. Although local recurrence after resection was not assessed in this study, previous findings from our institution have shown a local tumour recurrence rate of 3%.^14^ Importantly, local tumour progression after ablation is often amenable to repeat treatment,^7^ which may explain why higher recurrence rates does not translate into inferior OS. Furthermore, RFS has been shown to be an inadequate surrogate endpoint for OS in the setting of resectable CLRM.^15^ Our findings support the notion that tumour biology, rather than the radicality of local treatment, is the key determinant of survival after local therapy for CRLM.^16^

The COLLISION trial found no difference in survival after resection and thermal ablation of CRLM, using a noninferiority margin of hazard ratio 1.30.^7^ The trial randomized target lesions, and some patients had additional lesions suited for only one modality; 27% of procedures combined resection and ablation. Consequently, the COLLISION trial is not a patient-level head-to-head comparison of ablation vs resection, but it indicates that ablation of technically resectable lesions does not compromise survival.

Distinguishing true local recurrence from de novo metastases remains challenging. True local recurrence arises when a lesion is incompletely ablated or resected, allowing residual tumour cells to proliferate. Imaging cannot always differentiate residual disease from new metastases adjacent to the ablation zone. Similarly, local recurrences following R0 resections with wide margins may represent de novo metastases rather than residual disease. This distinction is clinically meaningful, as only true local recurrence is potentially preventable through technical refinement. Although local recurrence appears not to impact OS, repeat treatments have significant implications for patient burden and healthcare resources.

The incidence of local recurrence after ablation is expected to decrease with the implementation of ablation confirmation software, as demonstrated in the COVER-ALL trial.^17^ Furthermore, a recent consensus meeting is expected to establish a framework for future studies on ablation, including recommendations for ablation margins.

This study was conducted within a specific framework, as Oslo University Hospital is a high-volume centre for treatment of liver tumours in a centralized healthcare system. The institutional strategy focuses on minimally invasive, multimodal treatment with repeat procedures for recurrence. Consequently, it is challenging to isolate the effect of any single modality. It is also of limited value to evaluate only what the patients’ initial treatment should be. Even in randomized controlled trials the outcomes will be influenced by multimodal treatment for recurrence, and the complete treatment effect is determined by the entire management strategy rather than by one modality alone. Our findings suggest that within this framework the choice of resection versus ablation does not affect OS. Nevertheless, it represents an important clinical trade-off where the modalities differ in complication burden, recurrence patterns, need for retreatment, and the feasibility of retreatment.

This study has several limitations. The definition for parenchyma-sparing ablation was made post hoc and as such this does not constitute a true protocol-driven observational study. However, databases were prospectively maintained and are considered complete. Patients were treated at a single centre, which may reduce generalizability. Even with the exclusion of lesions > 3 cm, some tumours may not have been equally suitable for both modalities. Baseline differences between treatment groups persisted; patients undergoing resection had larger tumours, whereas patients undergoing ablation had higher ASA scores and more prior liver-directed treatments. There was a higher rate of ablations in the latter time interval. Although inverse probability weighting was used to mitigate selection bias, residual confounding cannot be excluded. Finally, some patients underwent multiple interventions and contributed data to both treatment groups. This overlap may appear counterintuitive, but the statistical approach accounts for repeated measurements. By including both initial and subsequent treatments, the analysis addresses which modality provides optimal outcomes at different points in the disease trajectory, rather than being limited to first presentations.

## Conclusions

Our results support a treatment approach that regards ablation as a noninferior alternative to resection when incorporated into a parenchyma sparing multimodal treatment strategy. Aligning with the COLLISION trial, these data suggest that all patients considered for local treatment for CRLM should be discussed in MDT meetings with expertise in both resection and ablation. In such a multimodal framework, both modalities can be used as first-line treatment and as treatment for recurrence and should be a matter of individualized approach to each patient.

## Supplementary Information

Below is the link to the electronic supplementary material.

Supplementary file1 (DOCX 1088 kb)
