# Supplementary material for: Long-Term Oncological Outcomes After Laparoscopic Resection and Thermal Ablation of Colorectal Liver Metastases Within a Parenchyma-Sparing Strategy
Source: Ann Surg Oncol. 2026 Apr 14;33(9):7833–41. doi: 10.1245/s10434-026-19621-1 (PMC13452845; doi:10.1245/s10434-026-19621-1)
Supplement: Supplementary file 1 [file 10434_2026_19621_MOESM1_ESM.docx]

## Supplementary material

**Supplementary Figure 1:** Overall survival in patients with no previous local liver treatment

P = 0.2741

**Supplementary Figure 2:** Overall survival after patient’s first liver treatment during the study interval. Stratified by tumour size 0-20 mm and 21-30 mm.

Ablation vs resection
Tumour size 0-20 mm: P = 0.7672

Tumour size 21-30 mm: P = 0.1738

**Supplementary Figure 3:** Progression-free survival after patient’s first liver treatment during the study interval. Stratified by tumour size 0-20 mm and 21-30 mm.

Ablation vs resection

Tumour size 0-20 mm: P = 0.0467

Tumour size 21-30 mm: P = 0.0005
